# On Movement as a Therapeutic Agent

**Published:** 1880-09

**Authors:** John K. Spender

**Affiliations:** London, Bath


ON MOVEMENT AS A THERAPEUTIC AGENT. By JOHN K. SPENDER, M. D., London, Bath.

The therapeutic doctrine of Rest has had so many prophets and bards, that it may seem daring to ask the question, whether it has not been pushed a little too far, or always used with discretion. The service done in this matter by the late Mr. Hilton cannot be overvalued, because, before 1861 (or thereabouts), there was no distinct appreciation of the physiological reasons of rest. It had been often applied without precision, and still more often in a halting and imperfect way. The enthusiasm which was the fruit of Mr. Hilton's teaching carried us almost out of the domain of pure unemotional science. The theory was not new, but it was put in the most attractive guise. Rest is first cousin to sleep; and if sleep can refresh and restore the healthy body, why may not rest undo some of the bad doings of disease? My present argument is not to disparage rest, but to plead for its more rational application. A remedy may be too benign, too quiet, almost too agreeable. It may be potent to subdue the slow workings of an insidious malady, but the healthy nutritional forces of the body require stir and exercise, anything but too much rest. Herein lies the danger—the real danger—that rest may not be left off at the proper time, and normal movement begun. I venture to ask, therefore, whether the therapeutic pendulum of rest has not swung rather too far.

It is indeed remarkable that there was no formulated doctrine of either rest or movement until within the last twenty years. As therapeutic agents, the words are not found in the index of Dr. J. Mason Good's *Study of Medicine* (1840); nor in that of Dr. Copland's *Dictionary of Practical Medicine* (1850); nor in that of Dr. J. Hughes Bennett's *Principles and Practice of Medicine* (1859). There is no index to South's *Translation of Chelius' Surgery* (1847), but I can find no allusion to the subject in this really grand work. It is admitted that, in an empirical sense, rest and movement have been employed since medicine became an art; but the scientific grounds for either one or the other were not always clearly grasped or opportunely carried out. The boundaries between the conditions which demand such opposite lines of treatment are sometimes not easily traced. How are we always to know when local symptoms have become so quiescent, that it is safe to undo the fetters of immobility and restraint, and tell a lame man that he may try to walk, or let a crippled hand begin to work? Pain is not an unerring guide. It is a fact in clinical medicine, that rheumatic neuralgia is sometimes worst when muscles are most rested; for a rheumatic person may awake after a night of good sleep with limbs stiff and painful, which symptoms distinctly pass away after moderate exercise and warm food. In like manner, surgical rest may continue too long.

Take the case of an acutely inflamed joint. Under the discipline of enforced rest, the synovial effusion becomes gradually absorbed; the classical signs of inflammation pass away; and when all danger of relapse has finally subsided, an organ of locomotion ought surely to be allowed gradully to return to its duty. But it is the fashion to confine an injured joint so long in wrappings and encasements of an immobile kind, that there is a risk of *quasi*-fibrous ankylosis, relaxation of ligaments, rigidity of tendons, and commencing atrophy of neighboring muscles. Now, in order to prove that these are not imaginary evils, I will relate in the briefest manner three cases selected from several more of a similar kind.

(*a.*) In March 1869, an elderly lady showed me her right knee, enveloped in splints and bandages. Towards the end of 1868, there had been an injury, the nature of which was obscure. She thought that she had struck or twisted the knee. There was little effusion into the joint, but the lower end of the vastus muscle seemed lifted from the femur as by a distended bursa. Hence this muscle acted at a disadvantage, and could not properly extend the leg. There was no sensation which could be called pain; but the stiffness and general uneasiness in the knee disabled her from walking, and *these symptoms supplied the reason why the joint had been kept so long at rest.* The leg was wasted, and there was a general weakness of the whole limb.

(*b.*) In October 1877, a gentleman under thirty years of age consulted me, and gave the following history. In July 1876, while "walking almost at a run in the dark, he fell into an iron harrow, the two knees fitting into a square of the harrow, so that the iron missed the knee-joint and met the limbs on the muscle above and on the bone below." He was not concious of any immediate symptoms, and walked about as usual; but in a few days there came on stiffness, and then pain, the latter being never of great severity. Local means gradually subdued local troubles; and then followed a long period of "irritability" (to use my patient's own word) for which the professional treatment was that the foot was for nine months supported in a sling, the other end of which went round the neck; and at night the knee-joint was packed in splints to prevent movement during sleep. For several months he carefully abstained from bending the leg upon the thigh, because he was emphatically told that continued rest was necessary. One result of this management was that, on the least strain or blow, the joint suffered from new attacks of "irritability" and pain.

(*c*.) The wife of a tradesman fell down some steps at the Bristol Railway Station on July 25th, 1878, and fractured the radius of the right arm. The fracture was properly set, and the splints were removed at the end of August. A bandage was then applied for three weeks. On November 1st, a starched immobile apparatus was put on, and allowed to remain for four months. When this patient first came to me, at the end of last May, the hand was nearly useless, simply from atrophy of the muscles. The hand had been imprisoned too long, and no directions had been given to arouse functional activity after its liberation from imprisonment.

Now the memory of some of my readers may recall the practice of the late Mr. Hutton, and the lessons gathered from it by Dr. Wharton Hood. The various conditions of joints which came under Mr. Hutton's treatment were easily classified, and included a large number which were stiff and painful from injury, or had been kept at rest for the avoidance of pain either after some injury to themselves or to the soft parts around them. Summed up in a few words, Mr. Hutton's principle was the rupture of adhesions by manipulative force, guided by skillful leverage. We learned from him that the joints will bear much rougher usage than is sanctioned by professional tradition; but this is not the point now at issue. The sort of case which I have just illustrated stands apart from those which are treated with sudden and violent flexion and extension. In dealing with a joint which has not been materially damaged by inflammation, is not much enlarged, but is now weak mainly because the muscles are weak which ought to move it, a thorough and scientific shampooing (or *massage*) is our therapeutic watchword. The physiology of the joint has to be studied more than its anatomy. The joint is afraid of itself because it is not used; its volitional energies are "hidden under a bushel;" and the owner of it has, perhaps, been scared by the terror of what may happen if he presumed to walk even with the help of crutches. It is satisfactory to know that Dr. Sayre, who is the designer of such an ingenious plan for supporting and resting a diseased spine, distinctly says that, in the case of ankylosed limbs, there is "a time for motion as well as a time for rest." *

First, then, we satisfy ourselves that the joint-ends of two bones are not mortared together, and do not require any forcible methods for their separation. A patient may be put partially under the influence of chloroform if necessary, and the capability of a joint to be bent or extended will then be fairly tested, and we shall discover what checks there are to healthy movement. The passive play of a joint tells us that its mechanism is sound; but the question is, What are its vital activities? What can the patient do with it, and how far can he use it? In the three cases which I have briefly delineated, I could see nothing to hinder the limbs from being actively employed. They needed to be brought out of the prison in which they had been immured by professional swaths and bands, and stirred into a more quickened life. They were suffering just as the whole body would suffer if, when clearly recovered from acute disease, it were still compulsorily confined to the bed or the bed-chamber.

The simple plan of treatment which I have for years adopted is as follows: Imagine the knee to be the affected joint, and the foot should rest on a stool or block of wood just within a large shallow open bath, so that the knee is over nearly the center of the bath. A jug, holding about a pint of fluid, is filled with tepid water, and is turned upside down about three feet above the knee, so that the water falls with a splash on the joint; and this is repeated from six to twelve times. An attendant, sitting in front of the patient, then plants a hand on each side of the knee, and, with the thumbs meeting in front, the hands should be moved firmly up and down for eight or ten minutes. The pressure used should be equal and well sustained, not causing any uneasiness, not in the least rough, but such an union of firmness and gentleness as a practical manipulator will easily understand. The thumbs, while agents of moderate pressure themselves, may be made the fulcra for pressing and rubbing the back of the joint.

---

* *British Medical Journal* August 30, 1879. While writing this paper, the 14th volume of *St. Bartholomew's Hospital Reports* came under my notice, and I have read with pleasure Mr. Willett's paper on Manipulation in Surgical Treatment. The cases which he relates are interesting; but they are examples of the utility of "forcible movement," and, therefore, belong to a different class from my own cases.

At the end of the shampooing process, the whole joint ought to be dry and warm, and to be immediately wrapped in a covering of oiled-silk lined with wadding, which should be securely fastened and kept on for some hours.

By this easy plan, carried out regularly once or twice a day for several weeks, there is seldom any difficulty in restoring the torpid functions of non-ankylosed joints. Now and then it may be desirable to suspend the friction for two or three days, if the skin show signs of irritation; and in warm weather the impervious pad is scarcely necessary, and might cause an eruption of pustular acne. Medicated lotions or liniments are rarely prescribed; but now and then I introduce under the oiled skin wrap a piece of folded flannel soaked in a mixture of tincture of iodine (half an ounce), glycerine (half an ounce), and soft water (seven ounces). The early douchings are best done with tepid water; but this should be exchanged for cold water as soon as possible, on account of the greater glow and reaction which are afterwards obtained; and, during the summer months, cold water may be used from the first. In all cases, the local treatment should be supplemented by regular passive movements carefully and coaxingly executed, and never exciting pain and fatigue. Sometimes it is only timidity which hinders a patient from (say) pronating and supinating a hand, or flexing and extending an elbow; a group of muscles have to be taught anew. As the lower limbs bear the weight of the body, their voluntary exercise must be deferred until the patient regains confidence and acquires strength.

The next stage of treatment consists in the application of uniform and gentle support. A weak joint requires support when it no longer requires rest. This seems to be the key of the new and important devices of the American surgeons—prolonged pressure without prolonged rest.* The principle is not at all new. Fifty years ago, my father was curing chronic ulcers of the leg by means of firm equal pressure with a domette flannel bandage, telling his patients to walk about as usual. His practice was opposed to the almost universal surgical teaching of the day, reit-

* As a remedy (or preventive) of acute inflammation, pressure has been enthusiastically discussed by Mr. Furneaux Jordan and Mr. Sampson Gamgee.

erated since with a loudness and constancy almost overpowering;* such surgeons as Dr. Underwood and Mr. Chapman being rare exceptions to the rule. And yet it ought to have been clear that to lay up a limb in idleness admits of the healing of an ulcer by a degree of vital action, which is unable to maintain the permanence of that healing when the limb is restored to use. Similarly, when we have to treat a weak joint, the principle ought to flash upon us with unerring instinct; here is a case, we should say, for applying firm pressure and allowing moderate exercise. For the *ankle*, there is nothing better than an india-rubber bandage, put on from the base of the toes to the upper part of the leg. The bandage is far better than a so-called elastic anklet, because it supports all the muscles which move the ankle; it should make a gentle uniform pressure, without any sensation of squeezing, each fold slightly overlapping the previous one all the way up the limb. The *knee* should be enclosed during the daytime in an elastic support, *which should always be laced*, and which need not cost more than four or five shillings. The lacing allows the degree of support to be varied, according to the feelings of the patient and the amount of exercise he has to undergo. Equipped with this simple apparatus, the lower limb enjoys, as far as the upper part of the knee, a gentle and uniform pressure, which permits useful locomotion within restraining and salutary conditions.

The application of this principle to the *upper limb* is not difficult. A hand and wrist, when embraced by an elastic glove extending a little way up the arm, is competent to perform various feats of dexterous workmanship, which would otherwise soon cause aching and powerlessness. But what need is there for illustrating further such an obvious point of practice? If to be at work is the part which a healthy organ has to play, it is clear that too much rest cannot contribute to the perfection of that work, either in quantity or quality. That special condition which is called the "hysterical joint" is peculiarly amenable to the

* Take at random such a sentence as the following: "Rest is Nature's own remedy, for the application of which she takes no denial; and the practitioner of the healing art takes his cue from this great teacher. If he have an ulcer on the leg to cure, he enjoins rest, and lays up the limb accordingly." (*Medical Times and Gazette*, October 19th, 1867.)

vigorous and helpful treatment of douching and pressure. For the douching, the use of the Bath waters is much to be commended, as the dynamic stimulus of their heat maintains an after-glow, which is a sign of more vital energy. And hysteria, if it mean anything, means a lack of this energy—a crippled volition, a sluggish blood-stream, and a general undertone of life. The combination of firm pressure and moderate exercise fulfills, in this case, exactly what we ought to aim at: support to the mechanical apparatus of locomotion, and arousing and sustaining of vital function.

I heartily agree with what Mr. Roth has written about the importance of muscle-rubbing in infantile paralysis.* He insists that the superficial and deep layers of muscles should be acted upon successively: firm circular friction is made on the same spot with the ball of the thumb, from left to right and from right to left, about ten times each way; pressure should be used at the same time with the thumb—the thumb being helped, if necessary, with the other hand. The fact is, however, that we ought to vary our manipulations according to the age and sensitiveness of the patient, and the duration of his malady. Thus, we use in one case stroking (*effleurage*), and in another kneading (*pétrissage*); sometimes percussion (*tapôtement*) is most beneficial; and, now and then, it is best to trust to passive motion. The last three methods are particularly adapted to chronic inflammations. Hard kneadings crush and break up old hyperplastic tissue, and the detritus is passed into the lymph-stream; vascular congestions are emptied, and the vaso-motor nerves are mildly irritated. The infriction of medicated substances may be an occasional aid, and among the best are mild iodine ointment and the liniment of iodide of potassium.

Rest and movement are, therefore, complimentary to each other, both in physiology and in therapeutics. The analogy is as close as possible. Alternation of work and rest is the law of the human organism in health, and health could not be preserved without it. Disease may call for a prolongation of the element of rest; but it is a note of clinical insight to discover when the

---

* *British Medical Journal*, June 14th, 1879.

disease ends, or when sufficient health returns to justify the usual alternate rest and work. Continued work without rest could not be ; rest continued too long is not only conceivable, but it is the object of this paper to illustrate it as one of the present dangers of therapeutic surgery.—*British Medical Journal.*

---

IMPROVEMENT OF SAYRE'S TREATMENT FOR SPINAL CURVATURE.—Mr. Richard Davy, of London, believes he has an improvement on Dr. Sayre's method of tripod suspension in applying the plaster of Paris jacket in spinal caries. He places the patient in a hammock face downward, arms hanging through slits in the canvas. Extension may then be used or not, according to the views of the surgeon, and the plaster of Paris or other dressing leisurely applied, including the canvas. A free circulation of air is allowed access to the body and the dressing dries rapidly, the patient often sleeping during the time employed. After the drying is complete the spare canvas is trimmed and the patient literally takes up his bed and walks. After reviewing some of the other methods of treating spinal caries according to Sayre's plan, that is of providing an outside support of the body, relieving the weak spinal column, Mr. Davy concludes in favor of his own plan. Aside from the small expense and inconvenience involved, a hammock some times being extemporized from a stout sheet or long night-gown, he thinks suspension not always quite safe in spinal and especially cervical caries, and that in removing the dressing after it causes pain, it is removed too late to avoid mischief. He quotes liberally from the late John Hilton "On rest and pain," and asks if any surgeon after thoughtfully reading Lecure 5, can be ready to fearlessly suspend cases of cervical caries.—*Foreign Cor. American Practitioner.*

